# Marginal bone loss and associated factors in immediate dental implants: a retrospective clinical study

**DOI:** 10.1186/s40729-025-00602-0

**Published:** 2025-03-26

**Authors:** Federico Rehberger Bescós, Ángel-Orión Salgado Peralvo, Cintia M. Chamorro Petronacci, Dumitru Chele, Fabio Camacho Alonso, David Peñarrocha Oltra, Óscar Lado Baleato, Mario Pérez Sayáns

**Affiliations:** 1 Oral Medicine, Oral Surgery and Implantology Unit (Medoralres), Faculty of Medicine and Dentistry, University of Santiago de Compostela, 15782 Santiago de Compostela, Spain; 2 Department of Stomatology, Faculty of Dentistry, University of Seville, 41009 Seville, Spain; 3 Health Research Institute of Santiago de Compostela (IDIS), ORALRES Group, C/ Entrerrios S/N, 15706 Santiago de Compostela, Spain; 4 Oro-Maxillo-Facial Surgery and Oral Implantology Department, State University of Medicine and Pharmacy ‘‘Nicolae Testemitanu’’, Chisinau, Republic of Moldova; 5 Department of Oral Surgery, University of Murcia, Murcia, Spain; 6 Oral Surgery Unit, Department of Stomatology, University of Valencia, Valencia, Spain; 7 Research Methodology Group, Health Research Institute of Santiago de Compostela (IDIS), Santiago de Compostela, Spain; 8 Institute of Materials of Santiago de Compostela (iMATUS), Santiago de Compostela, Spain

**Keywords:** Dental implants, Dental implantation, Endosseous, Alveolar bone loss, Tooth socket

## Abstract

**Purpose:**

The aim of this study was to evaluate the marginal bone loss (MBL) over a follow-up period of up to 36 months in Immediate dental implants (IDIs), as well as the impact of various clinical variables on the MBL.

**Methods:**

IDIs placed in two surgical phases were evaluated. Implants were classified into bone loss (BL, exposed threads), bone remodeling (BR, crestal bone at the implant margin ± 0.1 mm), and bone overlapping (BO, bone above the abutment).

**Results:**

A total of 1,040 IDIs were inserted in 344 patients with a successful osseointegration rate of 98.9%. The average MBL at 2, 6, 12, 24, and 36 months was − 0.3 ± − 1.0 mm, − 1.1 ± -1.8 mm, − 1.4 ± − 1.8 mm, − 1.7 ± − 1.9 mm, and − 1.3 ± − 2.3 mm, respectively. In the Baseline-12-month period, 17.5% of the IDIs presented BL, 9% BR, and 73.5% BO. For the B1-12 month period, 19.8% presented BL, 10.7% BR, and 69.5% BO. Mixed regression models showed significant MBL overtime pre-loading (*p* < 0.0001), stabilizing at 8.5 months from implantation. Immediate mandibular implants had lower MBL (*p* = 0.0365). Post-loading, MBL was lower in the mandible (*p* = 0.0095) and positively influenced by abutment height and rotational abutments.

**Conclusions:**

The present study supports the clinical efficacy of the IDIs placement protocol with high survival rates and acceptable MBL. It is recommended to place bone level implants slightly below the crest to ensure the platform remains at an optimal depth during the initial bone remodeling phase post-implantation.

**Supplementary Information:**

The online version contains supplementary material available at 10.1186/s40729-025-00602-0.

## Background

The development of dental implants has changed the way dentists routinely treat partial and total edentulism. As the literature describes, the estimated survival rate of implants after 10 years is 96.4% [1–4]. Even in patients with cardiovascular, endocrine, or neurological disorders, the implant survival rate is like healthy individuals [5, 6].

Traditionally, following tooth extraction, it would take 4–6 months for the healing of the soft and hard tissues [7, 8]. This approach had a series of disadvantages such as prolonged treatment time, lack of fixed provisional restoration, and significant alveolar ridge reduction both in soft and hard tissues [9, 10]. To overcome these challenges, 4 stages for implant placement were proposed. Type 1., immediate post-extractional implants; type 2., 4–8 weeks post-extractional; type 3., 12–16 weeks post-extractional; and type 4., more than 4 months after extraction [11].

The immediate implant survival rate is like other timings for implant placement [12, 13], with advantages such as a limited number of procedures, satisfactory esthetic results, the possibility of immediate provisionalisation, and being minimally invasive [14]. Immediate and delayed implants have been the subject of numerous studies comparing their performance in terms of MBL. Most studies conclude that both types of implants present similar levels of long-term bone loss, although differences may depend on various factors, such as follow-up time and patient characteristics [15]. On the other hand, some studies suggest that immediate dental implants placement (IIP) has limited indications and is considered a more advanced surgery [14, 16]. Regarding advantages, IIPs offer important benefits, such as the preservation of peri-implant tissues, including the gingival architecture and the vestibular bone, which favors the reduction of bone resorption. This contributes to better aesthetics, with an optimized emergence profile for prosthetic restoration, and greater functional stability of peri-implant soft tissues, which improves clinical and aesthetic results compared to deferred implants [17]. Another advantage of IIP is the possibility to seal the socket with a healing cap or provisional crown, therefore maintaining the soft tissue architecture and the hard tissues [18, 19]. Therefore, the stability of peri-implant tissues plays a crucial role in peri-implants’ overall health [20].

In the past, 1.5 mm of bone loss at 1 year of functional loading was considered physiological, followed by a subsequent loss of 0.1 mm of bone per year [21, 22]. Recent studies suggest that bone loss of > 0.5 mm, in the first 6 months after loading could potentially lead to peri-implantitis [20, 23].

Several factors are associated with marginal bone loss (MBL), which can be classified as patient-related, surgical, or prosthetic [24]. Smoking, a history of periodontal disease, and poor oral hygiene have been related to MBL [25–28]. Likewise, surgical techniques and protocols can also affect marginal bone [24]. Another important factor contributing to MBL is the implant-abutment interface. The localization, stability, type, and presence of platform-switching are believed to influence the marginal bone [29–34].

Additionally, a significant aspect to consider is the timing of final abutment placement. The biological development starts with the implant exposure to the oral environment [35]. This supracrestal complex comprises connective tissue, junctional epithelium and sulcular epithelium [36]. The connective tissue situated 1 mm above the implant platform is considered to act as a biological seal for the bone. Therefore, the protection of this seal is imperative for marginal bone stability [37, 38]. It is thought that the multiple disconnections of the prosthetic abutment could potentially lead to MBL [39–43], which can be considered by the clinicians irrelevant [44–46]. However, it is also believed that even a slight remodeling of the marginal bone can lead to uncontrolled resorption [20]. The placement of IDIs with healing caps (without high healing abutments) behaves like implants in two surgical phases (or submerged), allowing the final abutment to be positioned ideally after complete soft tissue healing [47].

The aim of this study was to evaluate the MBL over a follow-up period of up to 36 months in a large sample of implant placed in fresh sockets. Additionally, the study aims to analyze the impact of various clinical variables such as location, abutment height, and the type of prosthesis on the MBL.

## Methods

### Study design

This observational retrospective study followed STROBE (Strengthening the Reporting of Observational Studies in Epidemiology) guidelines [48]. All procedures performed in this study were in accordance with the ethical standards of the institutional and/or national research committee and with the 1964 Helsinki Declaration and its later amendments or comparable ethical standards. The research protocol was approved by the ethics committee of the CEIm San Carlos Clinic Hospital (Madrid, Spain) (Approval no. 24/803-E). All patients signed a written informed consent form for the collection of their teeth for the study.

### Participants

The following study was conducted in a cohort of patients seen in a single dental medical center in northern Spain (Oviedo, Asturias, Spain). To be included in the study, they had to meet the following eligibility criteria:

#### Inclusion criteria

The study included patients of both genders of legal age who agreed to participate and signed the informed consent form. Eligible patients were those treated with IDIs between 2018 and 2021, who did not require simultaneous bone regeneration, and who had a control X-ray at the time of surgery as well as subsequent check-ups at 2, 6, 12, 24, and/or 36 months. Patients with active periodontal disease, pocket depths greater than 5 mm, or a plaque index higher than 20% were excluded from the study.

#### Exclusion criteria

The patients who met the following criteria were excluded: simultaneous bone regeneration, pregnant or lactating women; patients with no or less than one year of clinical follow-up; patients with severe systemic disease, ASA classification III or IV; patients with untreated or uncontrolled periodontal disease; patients with medical disorders that adversely affect bone metabolism, such as osteomalacia, Paget's disease of bone, hyperthyroidism, cancer (excluding non-melanoma skin cancer), or alcoholism; patients undergoing radiotherapy or chemotherapy treatment of the head or neck, or having undergone these treatments at least two years prior to surgery; and advanced oncology patients.

STROBE flow chart is available in Supplemental Materials to improve the clarity of patient selection and study flow (Suppl. Mat).

### Surgical and restorative protocol

All surgeries were performed under local anesthesia by the same surgeon (RBF). All implants were placed in the same surgical procedure as the tooth extraction (IIP). The prosthetic loading was not conducted earlier than 2 months after surgery in any cases. The implants were placed according to the Misch and Silic criteria [49]. All implants were placed in two stages, positioning the implant 0.5–2 apical mm to the adjacent buccal bone crest, in order to position it 4 mm below the future gingival margin. In the case of a complex tooth extraction, an intrasulcular incision was made in order to see the junction between the tooth and the alveolar bone, taking advantage of the same flap to place the implant. In these cases, scalpel blade no. 15, scalpel handle no. 3 and Supramid^®^ 5/0 self-absorbent suture were used. All the implants used were from the company Galimplant^™^ (Sarria, Lugo, Spain). The implants used in the study are the IPX model implants with hexagonal internal connection, manufactured by the company Galimplant™ (Sarria, Lugo, Spain). This implant model is made of grade IV titanium and features a macroscopic design that promotes primary stability in various situations. It features a conical body with a straight neck shape, no microthreads on the neck, grooves at the apex, and no apical hole. Additionally, it features a conical internal connection of the hexagonal Morse taper type at 11º and a single prosthetic platform. Microscopically, the IPX implants have a Nanoblastplus surface with an average roughness (Ra) of 1.7 μm and are composed of 99.9% TiO2. Platform switching in IPX implants refers to a design in which the diameter of the abutment is smaller than that of the implant platform. In this case, the abutment has a diameter of 2.85 mm at the platform level, while the implant platforms have nominal diameters of 4.0 mm, 4.5 mm, or 5.0 mm. The “switching platform” or its narrowing varies according to the size of the implant: for 4.0 mm implants, the difference is 0.575 mm (per lateral wall of the implant neck, measured from the abutment to the outermost part of the platform), for 4.5 mm implants it is 0.825 mm, and for 5.0 mm implants it is 1.075 mm. This design allows distance of the connection between the implant/abutment (bacterial zone) towards the area of the implant's union with the bone, as well as a favorable distribution of loads on the bone, promoting the preservation of bone tissue and the stability of the implant.

All patients attended a follow-up consultation 10 days after surgery where sutures were removed if used.

The second phase of surgery, that is, the exposure of the IDI for connection of the transmucosal abutment, was performed 2 months post-surgery. The impressions for the fabrication of the fixed prostheses were taken with open trays. The passive fit was checked with all prosthetic frameworks and the occlusal adjustment was performed on all of them. In all cases the prostheses were fitted 4–6 weeks after the second stage surgery.

### Radiographic evaluation of the MBL

Orthopantomographies were performed on all patients on the day of IIP (baseline) and at subsequent check-ups (2 months post-surgery; 6, 12 and 36 months after prosthetic loading). Measurements on the radiographs were conducted with iDixel software version 3DXD 3.1.6.11345 J, made by Morita^®^ Corp, (Kyoto, Japan).

**Fig. 1:**
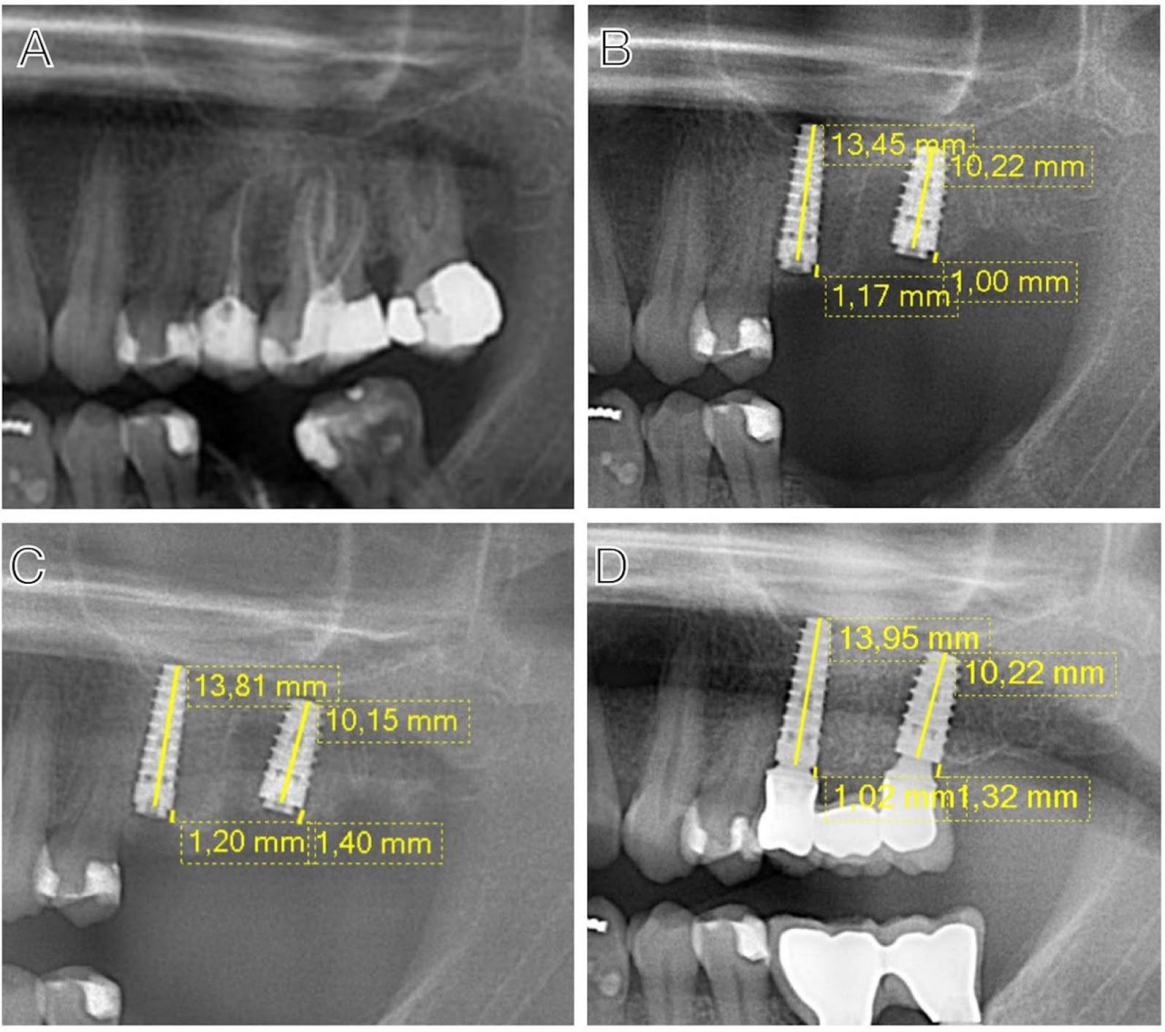
Measurements for the calculation of the MBL performed: **A** Preoperative evaluation; **B** immediate postoperative period; **C** 2 months after placement of the implants; **D** 36 month follow-up

Considering that orthopantomographies have a degree of distortion, the proportions were calculated based on the known length of the implants. To calculate the MBL, a single pre-calibrated examiner on a pilot sample of 50 implants (CMCP) performed all measurements, in a linear fashion from the base of the implant to the bone crest closest to the central axis of the implant, mesially and distally (Fig. 1).

Examiner calibration was performed before starting the study using 50 radiographs from 25 patients with late-placed implants, with the first radiograph taken on the day of surgery and the second two or more years after surgery. The calibration process was carried out in two stages: On the first day, all authors of the study met to clarify and agree on the specific measurement points. On the second day, the designated examiner measured all 50 radiographs. Measurements were performed on orthopantomographies taken at different time intervals. Reliability of measurements was assessed using the k statistic, which showed high intra-examiner reliability with a value of 0.91, confirming the consistency of measurements made by the single examiner. Regarding observer bias control, we implemented masking measures to prevent the examiner from knowing the follow-up time point, which could influence the assessment results. Furthermore, the assessment process was performed in a random manner, ensuring that the examiner had no access to patient information during the measurement. We also reinforced the validity of the process by duplicating measurements in several cases to obtain representative data and reduce any potential bias. There were no discrepancies or uncertainties regarding the measurement points throughout the process.

### Analyzed variables

Data was collected in a coded database (pseudonymized) and created for further statistical analysis.

**Fig. 2:**
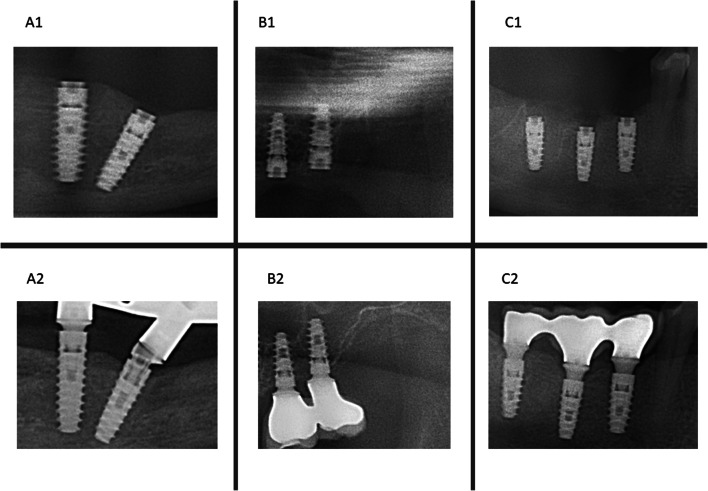
**A** BL example in IDI #33 (A1: March 2019- A2: September 2023); **B** BR example in IDI #26 (B1: November 2019- B2: January 2020); **C** BO example in IDI #46 (C1: January 2019-C2 January 2020)

The main variable is the MBL, which is measured at two distinct times, a pre-loading time (calculated from baseline B_0_ at post-implantation) and a post-loading time (calculated from baseline B_1_ at post-loading). In addition to quantifying the MBL, implants with follow-up at 12 months post-loading were classified according to Bernabeu-Mira et al. [50] into bone loss (BL, exposed spires), bone remodeling (BR, crestal bone over the implant margin ± 0.1 and bone overlapping (BO, bone over the abutment) (Fig. 2).

The process variables were: date of surgery, date of second stage surgery, date of prosthetic loading, implant height/diameter, implant position (maxillary/mandibular), final abutment height, date of check-ups after loading, measuring MBL on panoramic radiographs (at 2 months after surgery; 6, 12, 24, and/or 36 months post-loading), abutment type (rotational/antirotational), prosthesis type (cantilever/non-cantilever), implant survival (osseointegrated/failed). Clinical variables were gender, date of birth (age), comorbidities (yes/no), and tobacco use (yes/no/ex-smoker).

### Sample size calculation

To estimate the sample size, MBL in immediate implants at 12 months was used, with an expected reported mean of 1.5 ± 0.9 mm, and considering an alpha error of 5% and a statistical power of 90%, the necessary sample size was calculated. According to the established parameters, it was determined that 311 implants were required to obtain an accurate estimate of the MBL, guaranteeing adequate reliability and statistical significance in the results. The calculations were performed using the Epidat 4.2 software (SERGAS, Galicia, Spain).

Since our study included a total of 1040 implants, the sample size used is considerably higher than the minimum required, which significantly increases the statistical power of the study, allowing greater precision and robustness in the results obtained [51, 52].

### Statistical analysis

Statistical analysis was conducted with the IBM^®^ SPSS Statistics v.24.0 (IBM^®^ Corp., Armonk, NY, USA) software for Windows. For the description of the sample of categorical variables, frequency and percentage were used, and for quantitative variables, mean and standard deviation were used. Contingency tables analyzed with chi-square were created. Student's t-test or ANOVA and/or non-parametric analyses were used for comparison of means when normality was not verified.

A generalized additive mixed regression model that included a nested random intercept was fitted. This random effect considered that measurements taken over time belonged to the same piece, and that a patient could have more than one piece implanted. MBL to load and MBL post-load were considered as response variables. Explanatory variables included sex, implant location (jawbone/maxilla), abutment height and type, and time since IDI placement.

All differences where the value of *p* is ≤ 0.05 were considered statistically significant.

## Results

### Descriptive data

The final sample consisted of 1,040 implants placed in 344 patients, comprising 166 women (48.2%) and 178 men (51.7%), with an average age of 57.9 ± 13.5 years. Of these implants, 494 (47.5%) were placed in female patients and 546 (52.5%) in male patients. A total of 58.8% of the implants were placed in patients without comorbidities, compared to 41.2% in patients who had two or more systemic diseases. Non-smokers received 62.3% of the implants, smokers received 31.3%, and ex-smokers received 6.3%. Maxillary implants accounted for 63.7% of the total (n = 662 implants). The most common diameter was 4.0 mm (n = 508 implants; 48.8%), while the most frequent length was 12 mm (n = 404 implants; 38.8%). In terms of prosthetic components, 275 implants (26.4%) used anti-rotational abutments for single-unit prostheses, compared to 765 (73.6%) rotational abutments for multiple-unit prostheses. 19.3% of the splinted implants (n = 201 implants) were designed with cantilevered/extending prostheses. The most common abutment height was 1.5 mm (45.0%). A successful osseointegration rate of 98.9% was achieved, with only 11 failures (1.1%). Complete descriptive data can be found in Table 1.

**Table 1:** Descriptive data of the study sample

Variable	Specifications	N	%
Sex	Female	494 IDIs	47.5
		166 patients	48.2
	Male	546 IDIs	52.5
		178 men	51.7
Comorbidities	No	612 IDIs	58.8
	Yes	428 IDIs	41.2
Smoking	No	648 IDIs	62.3
	Yes	326 IDIs	31.3
	Ex-smoker	66 IDIs	6.3
Location	Maxillary bone	662 IDIs	63.7
	Jawbone	378 IDIs	36.3
Diameter DI (mm)	3.2	3 IDIs	0.3
	3.5	358 IDIs	34.4
	4.0	508 IDIs	48.8
	4.5	132 IDIs	12.7
	5.0	39 IDIs	3.8
Abutment type	Antirotational	275 IDIs	26.4
	Rotational	765 IDIs	73.6
Abutment height	1.0	124 IDIs	11.9
	1.5	468 IDIs	45.0
	2.0	120 IDIs	11.5
	2.5	96 IDIs	9.2
	3.0	201 IDIs	19.3
	4.0	31 IDIs	3.0
Implant height (mm)	6	16 IDIs	1.5
	8	113 IDIs	10.9
	10	254 IDIs	24.4
	12	404 IDIs	38.8
	14	215 IDIs	20.7
	16	30 IDIs	2.9
	18	8 IDIs	0.8
Osteointegration	Failed	11 IDIs	1.1
	Integrated	1029 IDIs	98.9
Cantilever	No	839 IDIs	80.7
	Yes	201 IDIs	19.3

Bold values mean that p-value is < 0.05

### Crestal position and marginal bone loss (MBL)

 The IDIs were placed at an average depth of 1.6 ± 1.1 mm subcrestally, ranging from 2.5 to 5.87 mm. MBL was assessed using two reference points: from the surgical procedure (Baseline: Bo) and from the time of prosthetic loading (At loading: B1) (Table 2). The mean MBL from Bo to 2 months was −0.2 mm (SD = 1.0), increasing to −1.1 mm (SD = 1.8) at 6 months. Over longer follow-ups, the mean MBL was −1.3 mm (SD = 1.8) at 12 months, −1.7 mm (SD = 2.0) at 24 months, and −1.3 mm (SD = 2.3) at 36 months.

**Table 2:** Marginal bone loss (MBL) during osseointegration period and after prothesis implantation. (*SD* = standard deviation)

Variable	N (IDIs)	Average (mm)	SD (mm)	Minimum (mm)	Maximum (mm)
MBL 6 months post-prosthetic loading (B_1_)	318	− 0.8	1.7	− 0.18	4.1
MBL B_1_ – 12 months	177	− 1.2	1.9	− 0.83	5.4
MBL B_1_ – 24 months	93	− 1.5	1.9	− 0.8	1.5
MBL B_1_ – 36 months	16	− 0.9	1.7	− 0.15	1.3

Bold values mean that p-value is <0.05

82 When considering MBL from B1 (loading) onward, the mean values were −0.3 ± 1.0 mm at 2 months, −1.1 ± 1.8 mm at 6 months, −1.4 ± 1.8 mm at 12 months, −1.7 ± 1.9 mm at 24 months, and −1.3 ± 2.3 mm at 36 months. For MBL from B1 to subsequent time points, the reported mean values were −0.8 ± 1.7 mm at 6 months, −1.2 ± 1.9 mm at 12 months, −1.5 ± 1.9 mm at 24 months, and −0.9 ± 1.7 mm at 36 months post-loading.

According to the classification of Bernabeu-Mira et al. [50], in the period B_0_−12 months, 17.5% of DIs had BL, 9% had BR and 73.5% had BO. For the period B_1_−12 months 19.8% had BL, 10.7% had BR and 69.5% had BO. There is a high correlation between these two variables (r = 0.492; *p* < 0.001).

### Relationship between MBL and clinical parameters

At 2 months, MBL was significantly greater in women (−0.3 ± 1.1 mm) than in men (− 0.2 ± 0.9 mm; *p* = 0.015). In the period B_0_−12 months, of the patients presenting with BL, 69.7% were female, while of those presenting with BO, 56.1% were male (*p* = 0.027). In the period B_1_−12 months, the percentage of females with BL was 62.9% and for implants with BO, males accounted for 61%.

Regarding smoking, a greater MBL at 2 months was observed in smokers (− 0.4 ± 1.1 mm) compared to non-smokers (− 0.2 ± 0.9 mm; *p* = 0.009). However, these differences reversed at the 36-month measurements.

In terms of location, implants placed in the upper jaw showed greater MBL at 12 months (− 1.6 ± 2.0 mm vs. − 1.0 ± 1.4 mm; *p* = 0.03) and at 24 months (− 2.2 ± 2.3 mm vs − 1.2 ± 1.4 mm; *p* = 0.008). Regarding abutment type, anti-rotational abutments for single-unit prostheses showed significantly higher MBL at 12 months than rotational abutments for multiple units, − 2.0 ± 2.3 mm vs − 1.1 ± 1.4 mm (*p* = 0.001). Regarding abutment height, differences were observed at 6 and 12 months. Thus, MBL at 6 months was lower for 2.5 mm height abutments (− 0.1 ± 1.0 mm) compared to 1.5 mm abutments (− 1.4 ± 2.0 mm) (*p* = 0.015). The same was true for MBL at 12 months with 1.5 mm abutments (− 1.0 ± 1.4 mm) compared to 3 mm abutments (− 2.1 ± 2.4 mm; *p* = 0.04). Interestingly, MBL at 2 months was higher in cases without cantilevers (− 0.3 ± 1.0 mm vs − 0.1 ± 0.9 mm; *p* = 0.017). No statistically significant differences were found related to comorbidities, nor to the diameter or height of the implant placed (Table 3).

**Table 3:** Relationship between marginal bone loss (MBL) at different moments and clinical parameters

Variable	Specifications		Average (mm)	DS (mm)	95% CI	*p*-value
MBL 2 months	Sex					**0.015**
		Female	− 0.3	1.1	− 0.4 to − 0.2	
		Male	− 0.2	0.9	− 0.3 to − 0.1	
	Smoking					**0.009**
		No	− 0.2	0.9	− 0.3 to − 0.1	0.009
		Yes	− 0.4	1.1	− 0.5 to − 0.3	0.009
		Ex-smoker	− 0.2	0.7	− 0.3 to 0	
	Pillar height (mm)					**0.008**
		1	− 0.1	0.7	− 0.2 to 0	> 0.05
		1.5	− 0.3	1.0	− 0.4 to 0.2	> 0.05
		2	− 0.4	1.0	− 0.6 to − 0.2	> 0.05
		2.5	− 0.1	0.5	− 0.2 to 0	> 0.05
		3	− 0.1	1.0	− 0.3 to 0	> 0.05
		4	− 0.7	1.7	− 1.4 to 0	> 0.05
	Cantilever					**0.017**
		No	− 0.3	1.0	− 0.4 to − 0.2	
		Yes	− 0.1	0.9	− 0.2 to 0	
MBL 6 months	Implant height (mm)					**0.018**
		6	− 3.3	7.2	− 10.9 to 4.2	NA
		8	− 0.7	1.4	− 1.2 to − 0.2	NA
		10	− 1.2	1.5	− 1.5 to − 0.8	NA
		12	− 1.2	1.7	− 1.5 to −0.8	NA
		14	− 0.9	1.4	− 1.2 to − 0.6	NA
		16	− 1.8	2.0	− 3.2 to − 0.4	NA
MBL 6 months	Abutment height (mm)					**0.027**
		1	− 0.9	1.8	− 1.7 to − 0.1	> 0.05
		1.5	− 1.3	2.0	− 1.6 to − 1.0	**0.015**
		2	− 0.7	1.2	− 1.3 to − 0.1	> 0.05
		2.5	− 0.1	1.0	− 0.5 to − 0.3	**0.015**
		3	− 1.0	1.6	− 1.4 to − 0.7	> 0.05
		4	− 1.3	2.0	− 2.9 to 0.2	> 0.05
MBL 6 months post OI	Implant height (mm)					**0.014**
		6	− 2.8	6.4	− 8.2 to 2.5	NA
		8	− 0.4	1.3	− 0.8 to 0	NA
		10	− 0.9	1.4	− 1.3 to − 0.6	NA
		12	− 1	1.5	− 1.2 to 0.7	NA
		14	− 0.7	1.4	− 1.0 to − 0.4	NA
		16	− 0.4	1.6	− 1.8 to 0.9	NA
MBL 12 months	Location					**0.03**
		Maxilla	− 1.6	2.0	− 1.9 to − 1.2	
		Mandible	− 1.0	1.4	− 1.3 to − 0.7	
MBL 12 months	Abutment type					**0.001**
		Antirotational	− 2.0	2.2	− 2.6 to − 1.4	
		Rotational	− 1.1	1.4	− 1.3 to − 0.8	
MBL 12 months	Abutment height (mm)					**0.014**
		1	− 1.7	1.7	− 17 to 13.6	> 0.05
		1.5	− 1.0	1.3	− 1.3 to − 0.8	**0.004**
		2	− 1.2	1.1	− 2.2 to − 0.1	> 0.05
		2.5	− 1.8	2.3	− 4.2 to 0.6	> 0.05
		3	− 2.1	2.4	− 2.8 to − 1.4	**0.004**
		4	− 1.6	2.0	− 3.4 to 0.3	> 0.05
MBL 12 months post OI	Location					**0.03**
		Maxilla	− 1.5	2.0	− 1.9 to − 1.1	
		Mandible	− 0.9	1.7	− 0.4 to − 0.2	
MBL 12 months post OI	Abutment type					** < 0.0001**
		Antirotational	− 2.0	2.5	− 2.7 to − 1.3	
		Rotational	− 0.9	1.4	− 1.2 to − 0.7	
MBL 12 months post OI	Abutment height					**0.01**
		1	− 1.7	1.3	− 13.4 to 10	NA
		1.5	− 0.9	1.3	− 1.1 to − 0.7	NA
		2	− 0.2	1.5	− 1.6 to 1.2	NA
		2.5	− 1.8	1.4	− 3.6 to 0	NA
		3	− 2.3	2.4	− 3 to − 1.6	NA
		4	− 1.1	3.9	− 5.2 to 3.0	NA
MBL 24 months	Location					**0.008**
		Maxilla	− 2.2	2.3	− 2.9 to − 1.6	
		Mandible	− 1.2	1.4	− 1.6 to − 0.8	
MBL 36 months	Smoking					**0.018**
		No	− 2.8	2.3	− 0.4 to − 0.2	**0.019**
		Yes	− 0.5	0.3	− 3.6 to 2.6	> 0.05
		Ex− smoker	0.4	0.9	− 0.6 to 1.4	**0.019**
MBL 36 months	Abutment height					**0.025**
		1	0.7			NA
		1.5	− 2.0	1.8	− 3.7 to − 0.3	NA
		2				NA
		2.5	0.2	0.9	− 1.0 to 1.3	NA
		3	− 0.9	2.6	− 24.5 to 22.6	NA
		4	− 6.7			NA
MBL 36 months post-loading	Smoking					**0.018**
		No	− 1.9	1.8	− 3.4 to 0.4	**0.047**
		Yes	− 0.7	0.1	− 1.4 to 0	> 0.05
		Ex-smoker	0.2	0.8	− 0.7 to 1.1	**0.047**

Bold values mean that p-value is < 0.05

(*CI* = confidence interval; *NA* = not applicable; *OI* = osseointegration)

Moreover, MBL showed a positive correlation at all different times, with a very strong correlation between MBL at 6 months and MBL at 24 months (Spearman r = 0.853; *p* < 0.0001). A strong correlation between age and MBL at 36 months was also reported (Spearman r = 0.714; *p* = 0.002). Linear regression models established that for each year of age increment, MBL increased by 0.098 mm (95% Confidence Interval CI − 0.151 to −0.045 mm; *p* = 0.001).

### Multivariate analysis of MBL

Generalized additive mixed regression models were constructed considering the MBL pre-loading/post-loading over time with various covariables. For the pre-loading model, a significant MBL overtime was estimated (*p* < 0.0001) (Fig. 2). The reduction in marginal bone level continued until 8.5 months after IIP, after which it stabilized.

It was determined that immediate mandibular implants showed lower MBL with an SE equal to 0.153; (95% CI 0.010 to 0.297; *p* = 0.0365). No significant effects of gender or abutment height were observed (Table 4).

**Table 4:** Generalized additive mixed regression models

I. PreOI Model							
Component	Term	Estimate	Std Error	t-value	CI 95%	*P-*value	Sig.
A. Parametric coefficients	Intercept	− 0.736	0.128	− 5.760	(− 0.987, − 0.486)	0.0000	***
	Sex: Men	− 0.030	0.076	− 0.394	(− 0.179, 0.119)	0.6935	
	DI site: mandible	0.153	0.073	2.093	(0.010, 0.297)	0.0365	*
	Abutment height (mm)	0.006	0.049	0.118	(− 0.089, 0.101)	0.9058	
Component	Term	**edf**	**Ref. df**	**F-value**		**p-value**	**Sig.**
B. Smooth terms	Time (months)	3.375	3.375	26.138		0.0000	***

II. PostOI Model							
Component	Term	Estimate	Std Error	t-value	CI95%	*P*-value	Sig.
A. Parametric coefficients	Intercept	− 1.318	0.371	− 3.554	(− 2.045, − 0.591)	0.0004	***
	Sex: Men	− 0.234	0.133	− 1.761	(− 0.494, 0.026)	0.0787	
	DI site: mandible	0.339	0.130	2.603	(0.084, 0.594)	0.0095	**
	Abutment height (mm)	0.087	0.085	1.017	(− 0.080, 0.254)	0.3096	
Component	Term	**edf**	**Ref. df**	**F-value**		***P*-value**	**Sig.**
B. Smooth terms	Time (months)	2.128	2.128	1.224		0.3944	

III. PreOI Model with type of abutment							
Component	Term	Estimate	Std Error	t− value	CI95%	*P*-value	Sig.
A. Parametric coefficients	Intercept	− 1.070	0.244	− 4.392	(− 1.548, − 0593)	0.0000	***
	Sex: Men	− 0.033	0.076	−0.435	(− 01.82, 0.116)	0.6638	
	DI site: mandible	0.136	0.074	1.837	(− 0.009, 0.280)	0.0664	
	Abutment height, (mm)	0.100	0.077	1.300	(− 0.051, 0.250)	0.1937	
	Abutment type: rotational	0.213	0.131	1.634	(− 0.043, 0.469)	0.1025	
Component	Term	**edf**	**Ref. df**	**F-value**		***P*-value**	**Sig.**
B. Smooth terms	Time (months)	3.378	3.378	26.212		0.0000	***

IV. PostOI Model with type of abutment							
Component	Term	Estimate	Std Error	t-value	CI95%	*P*-value	Sig.
A. Parametric coefficients	Intercept	− 3.256	0.769	− 4.237	(− 4.763, − 1.750)	0.0000	***
	Sex: Men	− 0.242	0.132	− 1.828	(− 0.501, 0.017)	0.0681	
	IDI site: mandible	0.283	0.131	2.157	(0.026, 0.539)	0.0314	*
	Abutment height (mm)	0.441	0.152	2.892	(0.142, 0.739)	0.0040	**
	Abutment type: rotational	0.762	0.257	2.970	(0.259, 1.265)	0.0031	**
Component	Term	**edf**	**Ref. df**	**F-value**		***P*-value**	**Sig.**
B. Smooth terms	Time (months)	2.091	2.091	1.142		0.4028	

Bold values mean that p-value is < 0.05

*DI* = dental implant; Std Error = Standard Error; Sig = Significance; edf = effective degrees of freedom; Ref. df = reference degrees of freedom)

*Signif. codes*: 0 <  = '***' < 0.001 < '**' < 0.01 < '*' < 0.05

**Fig. 3:**
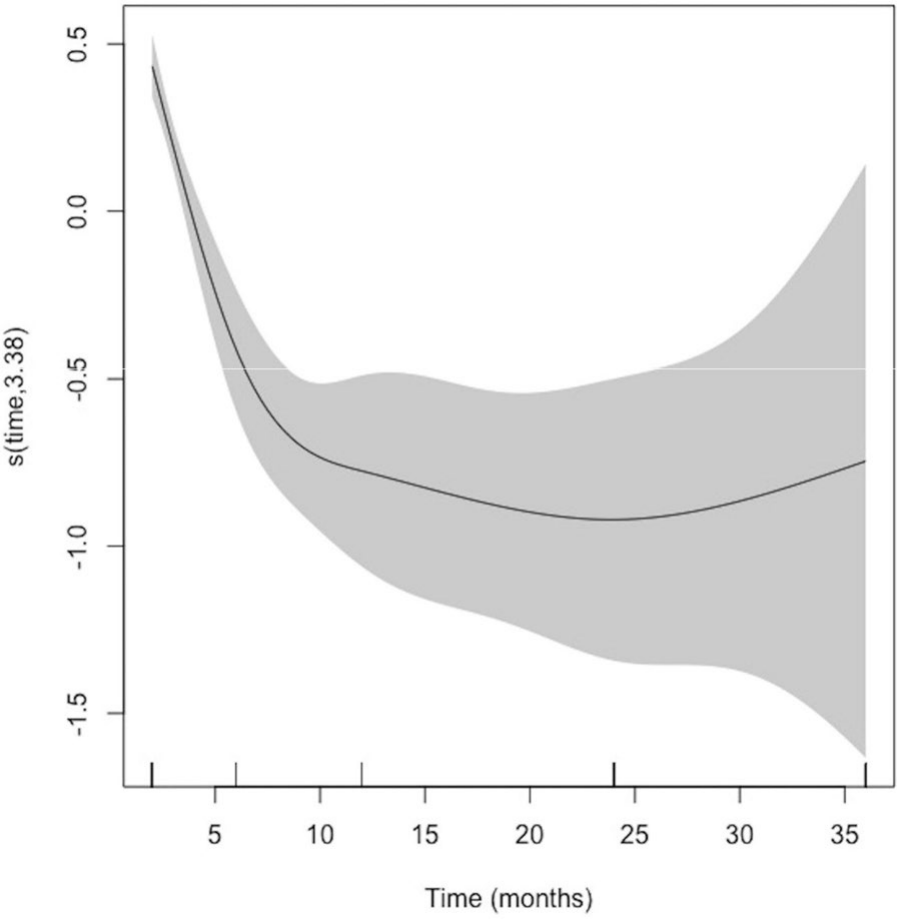
Time effect on marginal bone loss pre-osseointegration (PreOI)

For the post-loading MBL model, bone loss was not associated with time (*p* = 0.3944) (Fig. 3). The covariate 'jaw' was significant, with lower MBL in the mandible than in the maxilla, with an SE = 0.339; (95% CI 0.083 to 0.594; *p* = 0.0095) (Table 4).

**Fig. 4:**
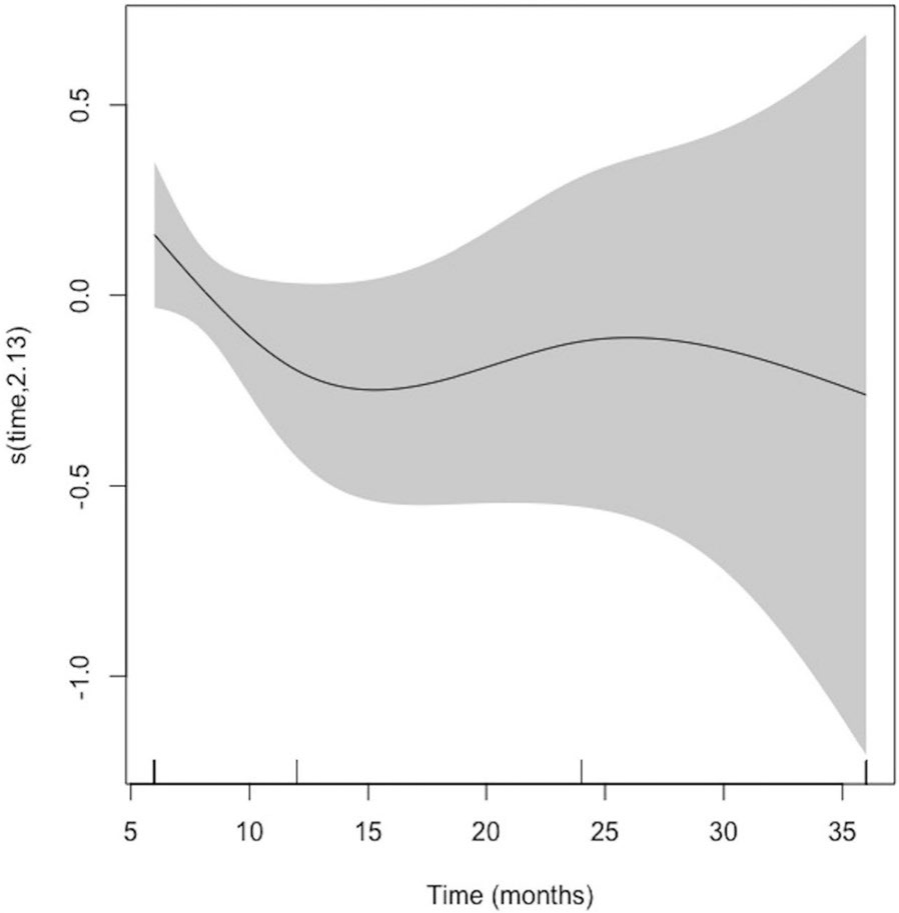
Time effect on marginal bone loss post-osseointegration (PostOI)

Models were also constructed to evaluate the impact of abutment type (single-unit or multiple-unit prostheses). For the pre-loading model, a significant MBL overtime was observed (*p* < 0.0001) with no other associated covariables. However, evaluating the post-loading MBL, a time independent MBL was noted (*p* = 0.4028), with lower MBL in the mandible (SE = 0.283; 95% CI 0.025, 0.539; *p* = 0.031), but also demonstrated a positive impact related to the abutment height (SE = 0.441; 95% CI 0.142 to 0.740; *p* = 0.0040) and rotational abutments (SE = 0.762; 95% CI 0.259, 1.265; *p* = 0.0031) (Fig. 4).

## Discussion

Scientific evidence has shown that immediate DIs have similar survival rates to those described in other implant insertion protocols. Specifically, several systematic reviews and meta-analyses observed survival rates inIDIs of 93.80% (n = 371 implants) versus 95.88% (n = 194 DIs) [53]–100% in implants inserted early in follow-up periods of 12 to 120 months, with no statistically significant difference between the two [53]. Definitions of "early implant insertion" varied between studies from 3–5 weeks to 6–8 weeks. These rates improved when analyzing implants inserted in maxillary anterior sites (98.2% vs. 100%, respectively; *p* = 0.480), although the follow-up periods were shorter (8 to 24 months) [54]. However, other authors have found significant differences when comparing immediate versus delayed (i.e. > 6 months after tooth extraction) implants after previous alveolar preservation. Specifically, 94.9% (n = 233 DIs) vs. 98.9% (n = 240 implants), respectively (*p* = 0.02) [55] at 12–96 months follow-up. Considering the above data, the success rates of 98.9% at 36 months follow-up described by the authors are in line with these results considering the period studied, however, the sample size included is significantly higher (n = 1040 implants).

Regarding the MBL, the results are very heterogeneous, with losses ranging from −0.04 to − 0.54 mm at 12—24 months follow-up [54]. In general, higher MBLs are observed in immediate DIs than in early protocols, namely − 0.03 [54] to −0.14 mm higher [53]. However, when compared with deferred implants, the results are very heterogeneous, showing gains in favor of immediate implants of + 1.23 mm (*p* = 0.04) when bone regeneration is not performed simultaneously, while when it is performed, no significant differences are observed [55]. Authors attribute this to the fact that the measurements in most of the studies are taken at the time of implantation, so the first implant-bone contact is far from the implant platform in the coronal-apical direction. After several months of healing, bone formation takes place in this area, resulting in greater bone gains. In contrast, in delayed-inserted implants, the implant is surrounded by bone [55–57]. For this reason, it would be prudent to compare the MBL between different protocols when bone remodeling has occurred (at the time of definitive fixed prosthesis connection), as in the present study where the MBL was studied with reference to the time of immediate implants insertion as well as after prosthetic loading. Thus, for the same point, for example, at 36 months after implant insertion, the authors observed an MBL of − 1.3 mm while at 36 months after loading it was lower (− 0.9 mm).

Bone loss during the healing phase, prior to prosthetic loading, has been studied in retrospective studies like the present one, with similar measurement protocols and a mean loss of − 0.5 mm [58], in line with the mean MBL obtained at the time of the second phase of our patients, which was − 0.2 mm ± − 1.0 mm. This early bone loss of less than − 0.5 mm is considered acceptable, however, a higher MBL would significantly increase the risk of progression over time towards peri-implantitis [59], reflecting the positive correlation of MBL with respect to all time periods evaluated, with a very strong correlation between MBL at 6 months and MBL at 24 months (Spearman r = 0.853; *p* < 0.0001). The regression models estimate a progressive MBL (on average − 0.3 mm in the first two months and − 1.1 mm at 6 months) up to 12 months (− 0.736 mm), from which time there is stability—even with some improvement—from 24 months onwards.

This increase in MBL at 6 months may be explained by the fact that the disruption of the epithelial junction of the peri-implant mucosa by abutment disconnection produces a secondary MBL. A recent meta-analysis by Koutouzis et al. [60] revealed that implants that underwent multiple abutment disconnections/reconnections had 0.19 mm more MBL. In this regard, Abrahamsson et al. [61] studied the effect of abutment disconnection/reconnection on peri-implant tissues in animal models, observing that in the test group the marginal bone level was located 1.5 mm from the implant-abutment connection, i.e., approximately 0.7 mm more apical compared to the control group (*p* < 0.05). The distance from the mucosal margin to the implant-abutment connection was 1.02 mm in the test group and 2.54 mm in the control group, i.e., there was a mucosal recession of approximately 1.5 mm in the test group, resulting in an apical insertion of the junctional epithelium. Repeated connections-disconnections were caused by first placing the healing abutments and, subsequently, the impression transfers, as well as intermediate trials of the fixed prostheses and, finally, their definitive connection. Reflecting this, the multivariable model observed a significant MBL over time (*p* < 0.0001). The reduction in marginal bone level continued until 8.5 months after immediate implant placement, after which it stabilized as the implant had already been loaded.

On the other hand, some research suggests that MBL is independent of the type of prosthetic rehabilitation and the timing of loading. The association with lower MBL in DIs with rough surfaces, platform change and a subcrestal location seems to have been demonstrated in previous work [62, 63]and are control factors in the present study using the same implant system. More recent analyses also show lower survival rates in implants loaded immediately compared to conventional loading, i.e. after the osseointegration period, but no other differences in terms of bone remodeling, probing depth or peri-implant mucosal level [64]. Regarding abutment type, the results of the regression models showed that anti-rotational abutments used in single-tooth prostheses had a significantly higher MBL at 12 months after loading (*p* < 0.001). In contrast, greater crestal bone gain is seen in taller abutments and rotational abutments (*p* = 0.003). This has been confirmed by recent meta-analyses showing a strong association between abutment height and MBL, with heights greater than 2 mm proving to be an important protective factor in peri-implant bone maintenance [65].

Overall, MBL is statistically higher in smokers after functional loading, but not in the first months after surgery. Previous systematic reviews leave no doubt about the influence of tobacco use and the risk of implant failure in general [66] and peri-implantitis in particular [67], so that a rebound effect could be observed in these patients after further follow-up. The implants placed in the maxilla have also undergone a higher mean bone remodeling than those placed in the mandible (*p* < 0.05), as has also been confirmed in previous studies [68].

MBL, as well as the survival rates of the implants, do not differ significantly from those implants inserted early or deferred. Thus, they should be considered whenever the patient's characteristics indicate them, as they reduce the chair time by more than half compared to deferred protocols [69] as well as the overall treatment time, with similar levels of patient satisfaction [70], and with a lower morbidity. This is because the other protocols involve a higher level of invasiveness by requiring the performance of one or two releasing incisions and a periosteal incision [54].

In the study of immediate implants, several methodological limitations and clinical considerations emerged as critical points that influenced the assessment and results. Methodological limitations included the measurement of the MBL using orthopantomographs, although this was compensated for by using the same device and calculating the distortion in each implant. In addition, the lack of recording the average number of cigarettes smoked daily by smoking patients and the omission of data on probing depth and the presence of mucosal recessions limit a complete understanding of the morphological and esthetic changes in the peri-implant soft tissues.

The SARS-CoV-2 pandemic exacerbated these difficulties by making it difficult to closely follow patients’ post-insertion of implants during 2019–2020, affecting the continuity and accuracy of the data collected.

In clinical terms, the influence of using two-stage protocols versus simultaneous abutments is debatable, as well as the potential benefit of individualized temporaries or healing caps to protect the alveolus and promote healing. In addition, the choice of biomaterials for the peri-implant gap emerges as a critical factor that could significantly impact the biological response and osseointegration of the implant.

As a retrospective study, the data is based on patients who have already been treated, which may introduce bias in the selection of cases. Although a considerable number of implants were included, the sample size may not have been large enough to detect significant differences in subgroups of patients, such as those with comorbidities or specific conditions. Factors such as the systemic health of the patient, the control of diseases like diabetes, oral hygiene, or the use of medications that affect bone healing may not have been adequately controlled, which could have influenced the results of MBL.

Although the results were collected over a period of 3 years, a longer follow-up may be necessary to accurately assess the long-term effects of immediate implants, such as stability at 5 or 10 years. The lack of a thorough evaluation of the peri-implant soft tissues, such as the measurement of keratinized mucosa, may limit the ability to fully assess the clinical and aesthetic outcomes of immediate implants. Finally, the evaluation of the results was performed by a single examiner, which could introduce bias. However, as a contingency plan, good calibration was established beforehand to minimize these risks. It is also recognized that there may be difficulty in accurately identifying the alveolar crest in some radiographs, which could influence the assessment of MBL.

As recommendations for further research, it would be interesting to know whether the administration of preventive antibiotics influences MBL as recent studies have linked the use of antimicrobials other than amoxicillin to higher rates of implant failure (71,72). On the other hand, it is recommended that when reporting MBL data in the future, measurements should be given from the time of implantation and from the time of functional prosthetic loading, otherwise measurements may be over or underestimated.

## Conclusions

The present study supports the clinical efficacy of immediate implant placement protocol with high survival rates and acceptable MBL. Regarding the latter, the insertion of implants bone level about 3 mm infracrestal should be considered to ensure a subcrestal implant platform position during bone remodeling during the first months after implantation. The insertion of immediate implants in the jaw compared to the maxilla, the abutment height and rotational abutments demonstrated a positive impact over the MBL.

## Supplementary Information

Supplementary material 1

## Data Availability

No datasets were generated or analysed during the current study. The datasets used and/or analysed during the current study are available from the corresponding author on reasonable request

## Abbreviations

IDIs: Immediate dental implants
MBL: Marginal bone loss
