# Supplementary figures and images for: Marginal bone loss and associated factors in immediate dental implants: a retrospective clinical study
Source: Int J Implant Dent. 2025 Mar 26;11:25. doi: 10.1186/s40729-025-00602-0 (PMC11947403; doi:10.1186/s40729-025-00602-0)

## Flow Diagram

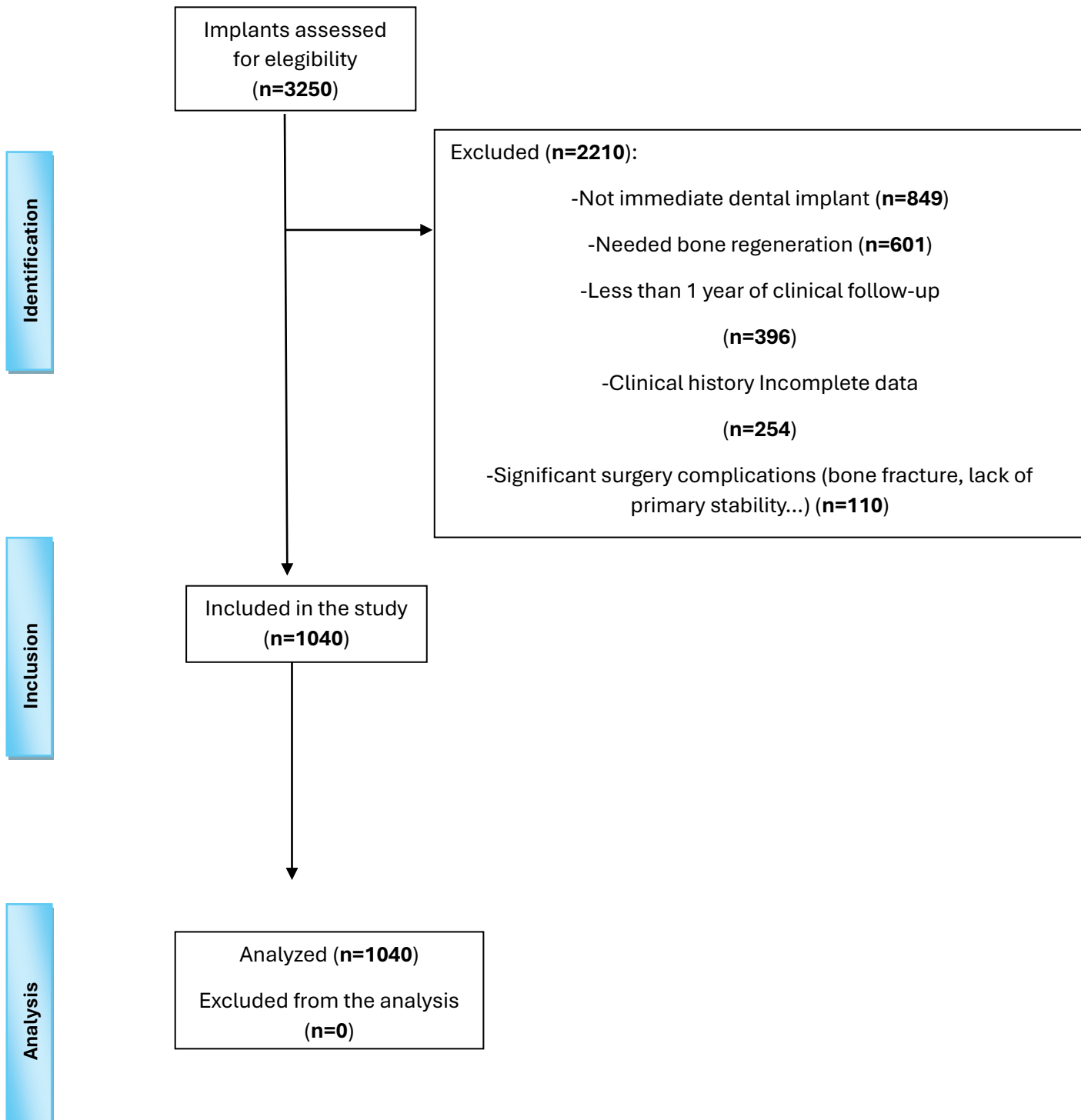

Supplement: Supplementary file 1 — Supplementary material 1 [file 40729_2025_602_MOESM1_ESM.pdf]
